# The influence of preoperative urodynamic parameters on clinical results in patients with benign prostatic hyperplasia after transurethral resection of the prostate

**DOI:** 10.1007/s00345-023-04656-w

**Published:** 2023-10-20

**Authors:** Jiyao Yang, Hongde Song, Hui Zhan, Mingxia Ding, Ting Luan, Jian Chen, Hairong Wei, Jiansong Wang

**Affiliations:** Urology Department, The Second Affiliated Hospital, Kunming Medical University, Kunming, Yunnan China

**Keywords:** Benign prostatic hyperplasia (BPH), Clinical outcomes, Influence factors, Transurethral resection of the prostate (TURP), Urodynamic analysis

## Abstract

**Purpose:**

To identify the urodynamic parameters affecting the clinical outcomes of transurethral resection of the prostate(TURP) surgery for patients with benign prostatic hyperplasia(BPH) by multifactor analysis and establish a regression model with diagnostic values.

**Methods:**

The medical records of patients who underwent TURP surgery for BPH between December 2018 and September 2021 were collected from the urology department of the Second Affiliated Hospital of Kunming Medical University, Kunming, China. The patients’ clinical data and urodynamic parameters were collected before surgery. The urodynamic parameters affecting surgical efficacy were identified by multifactor analysis, and a regression model with diagnostic values was established and evaluated.

**Results:**

A total of 201 patients underwent TURP, of whom 144 had complete preoperative urodynamic data. Each urodynamic factor was subjected to multifactor analysis, and the bladder contractility index (BCI), bladder outflow obstruction index (BOOI), bladder residual urine, and bladder compliance (BC) were found to be independent influence factors on the efficacy of TURP in patients with BPH. The diagnostic value of the regression model was analyzed by receiver operating characteristics (ROC) analysis, and it was found that the AUC = 0.939 (95% CI 0.886–0.972), for which the sensitivity and specificity were 95.19% and 80%, respectively.

**Conclusions:**

The regression model had high diagnostic sensitivity and specificity in predicting the efficacy of surgery, and the diagnostic value was higher than that of individual urodynamic factors. Therefore, BCI, BOOI, bladder residual urine, and BC should be considered as independent influence factors on the efficacy of TURP surgery for BPH.

## Introduction

Transurethral resection of the prostate (TURP) is one of the dominating therapies for patients with benign prostatic hyperplasia (BPH) with severe lower urinary tract symptoms (LUTS), and most patients experience significant improvement in symptoms postoperatively. However, in some patients, LUTS do not resolve after surgery. In a randomized controlled study with a mean follow-up of 2.8 years, John H. Wasson et al. reported surgical failure in approximately 9.2% patients after TURP (23/249) [1]. In another randomized controlled study by Robert C. Flanigan et al., in which 280 BPH patients who received TURP and were followed up for more than 5 years, a 10% failure was reported [2].

Inadequate understanding of the patient’s indications for surgery can lead to surgical failure. Although indications for TURP surgery have been clarified in various clinical guidelines and by expert consensus, whether a patient requires TURP surgery is mainly based on assessments of the patient’s symptoms and/or complications [3]. Subjective assessments of patient symptoms and the diversity of causes of complications may promote the recommendation of TURP surgery for some patients whose outcomes do not fulfill the predictions that were made according to the surgical indications, thus resulting in patient dissatisfaction. Hence, there is an urgent need for more accurate clinical indicators to assist urologists in selecting patients with BPH for TURP surgery, thereby improving clinical outcomes.

In recent years, researchers have applied urodynamic analysis to determine how to manage BPH and found a correlation between certain urodynamic parameters and the outcome of surgical treatment of BPH. For instance, Mauricio Plata et al. reported that BPH patients with detrusor underactivity (DU) were less likely to experience alleviated LUTS and improved quality of life after surgery and more likely to have urinary retention [4]. Yan Zhu et al. also reported that in patients with BPH causing bladder outlet obstruction combined with DU, the success rate of surgery decreased significantly [5].

However, multifactor studies comprehensively observing the effect of different urodynamic parameters on the efficacy of TURP are still lacking. Therefore, we intend to explore the correlation between different urodynamic parameters and the efficacy of TURP through multifactor analysis. We constructed a logistic regression model including multiple urodynamic factors and explored its diagnostic value in judging the efficacy of TURP through the ROC curve.

## Materials and methods

The medical records of patients who underwent bipolar TURP surgery for BPH performed by the same group of urologists between December 2018 and September 2021 were collected from the Department of Urology, Second Affiliated Hospital of Kunming Medical University, Kunming, China.

Inclusion criteria: (1) Patients who underwent TURP surgery with a clinical diagnosis of BPH; (2) patients aged ≥ 45 years; and (3) patients without serious surgery-related complications.

Exclusion criteria: (1) Patients with a pathologic diagnosis such as prostate cancer after surgery; (2) patients with neurological diseases, mental disorders, diabetes, or other systemic diseases that may affect urinary control; (3) patients with urethral stricture, bladder neck sclerosis, bladder stones, or other diseases that may cause bladder outlet obstruction; (4) patients with a history of prostate, urethra, or pelvic surgery; and (5) patients with incomplete examination data or follow-up data.

### Variables and outcomes

Preoperative clinical data were collected on variables, including age, International Prostatism Symptom Score (IPSS) score, quality of life (QOL) score, and history of urinary incontinence, urinary retention, diabetes mellitus, hypertension, smoking, and alcohol consumption. Preoperative urodynamic examinations were performed by the German Andromeda Ellipse urodynamic system, and all tests were performed in accordance with urodynamic quality control standards [6–8]. The urodynamic parameters that were included were as follows: residual urine, bladder compliance (BC), bladder outflow obstruction index (BOOI), bladder contractility index (BCI), maximum bladder capacity, maximum urine flow rate (Qmax), and unstable bladder contraction. The TURP procedure was performed by the same team of urologists for each patient. Patients were followed up by phone call or face-to-face conversation 3 months after surgery, and postoperative IPSS scores and QOL scores were collected during the follow-up visit. Effective surgery was established as the ratio of postoperative IPSS to preoperative IPSS at ≤ 0.5 and a postoperative QOL score higher than the preoperative QOL score by 3 points; otherwise, the surgery was considered invalid. A multifactor analysis was performed to identify urodynamic factors affecting surgical efficacy, and a logistic regression model with diagnostic value was developed and evaluated.

### Statistical analysis

IBM SPSS statistical software (Version 25.0, Chicago, IL, USA) was employed for data analysis. Data conforming to normal distribution was expressed as mean ± standard deviation, and data not conforming to normal distribution was expressed as median and quartiles. Univariate analysis was performed by the chi-square test, and multivariate analysis was performed by logistic regression. ROC curve analysis was performed using Medcalc 19.6.4 software. A *P*-value of less than 0.05 was considered to be statistically significant.

## Results

### Patient characteristics

**Fig. 1:**
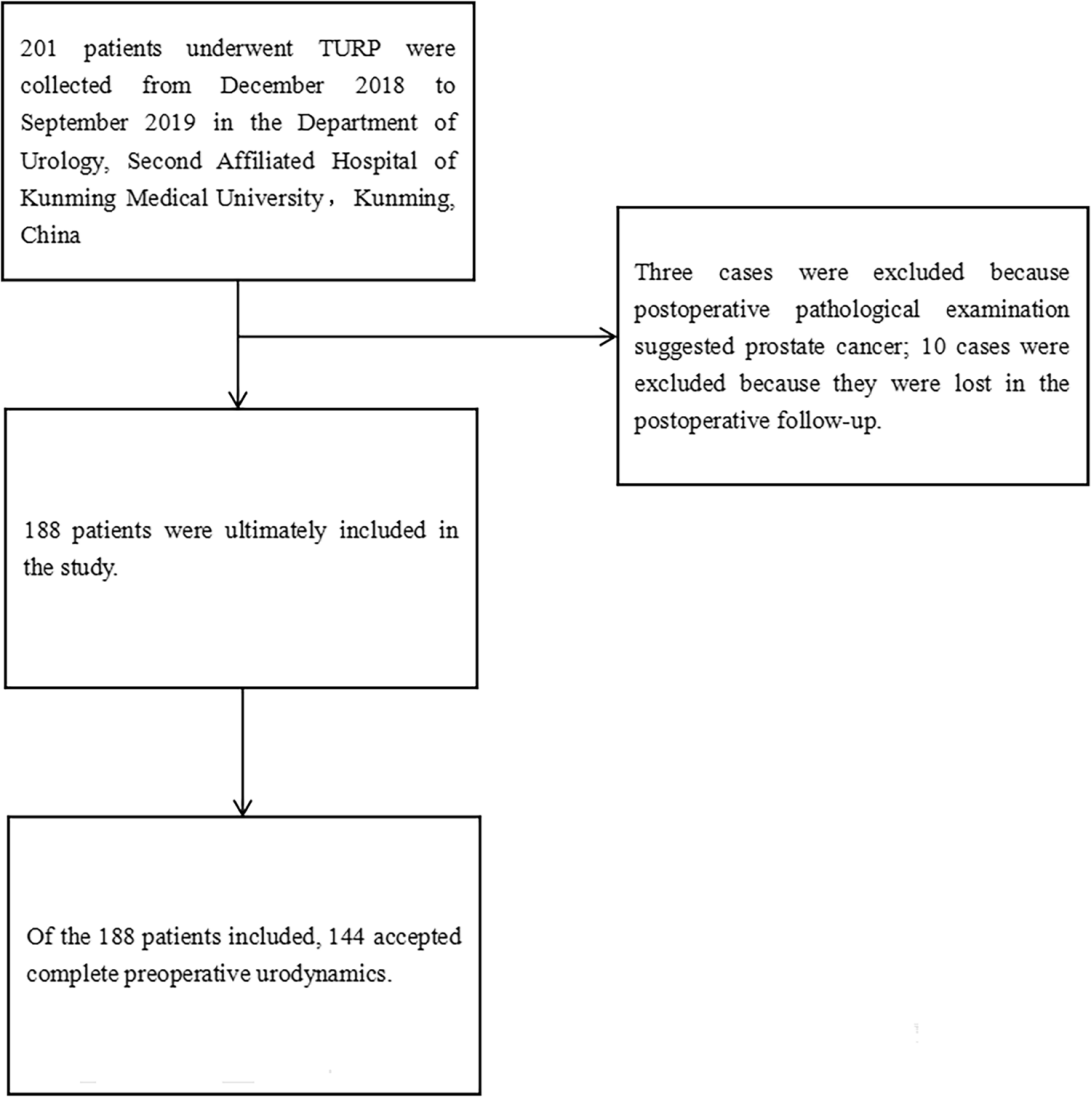
Flow chart of patient enrollment

A total of 201 patients were collected for this study, among which three cases were excluded when their postoperative pathological examination suggested prostate cancer, and 10 cases were lost during postoperative follow-up. Finally, 188 patients were included in this study, of which 144 patients had complete preoperative urodynamic examination data (Fig. 1). All patients were followed up at least once after surgery, with a median follow-up time of 5 months (3–22 months). Among the 188 patients, the median age was 67 years (interquartile range 61.25 to 74 years). In total, 75 (39.9%) had a history of urinary retention, 53 (28.2%) had a history of urinary incontinence, 46 (24.5%) had a history of diabetes mellitus, 87 (46.3%) had a history of hypertension, 109 (58%) had a history of smoking, and 77 (41%) had a history of alcohol consumption before surgery. According to the surgical efficacy criteria of TURP, 139 cases were judged effective and 49 cases were judged ineffective after surgery, for a total efficacy rate of 73.94%.

### Determination of predictors for the efficacy of TURP

The mean of the BCI for the 144 patients who underwent preoperative urodynamics was 91.11 ± 36.47; the mean of maximum bladder volume was 324.84 ± 97.48 ml; the median of Qmax was 7.05 ml/s (interquartile range 5 to 8.98 ml/s); the median of BC was 28.95 ml/cm H_2_O (interquartile range 12.32 to 40.97 ml/cmH_2_O); and the median of bladder residual urine was 15 mL (interquartile range 0–66 ml). The threshold of the BOOI was set at 40. After dichotomizing each urodynamic parameter by the mean, median, or threshold, a univariate analysis was performed, and the results showed BC (*p* < 0.001), bladder residual urine (*p* < 0.001), BOOI (*p* < 0.001), BCI (*p* < 0.001), and unstable contraction of bladder (*p* < 0.001) as the independent predictors affecting the postoperative outcome of TURP (Table 1).

**Table 1:** Urodynamic factors affecting the efficacy of TURP surgery (univariate analysis)

Types		Clinical Effectiveness		Efficient	^χ2^	p
		Ineffective group (n = 40 cases)	Effective group (n = 104 cases)			
Bladder compliance(ml/cmH_2_O)	≤ 28.95	36	36	50.00%	35.446	<0.001
	> 28.95	4	68	94.44%		
Bladder residual urine(ml)	≤ 15	9	64	87.67%	17.614	<0.001
	> 15	31	40	56.34%		
BOOI	≤ 40	33	29	46.77%	35.146	<0.001
	> 40	7	75	91.46%		
BCI	≤ 91.11	37	36	49.32%	38.726	<0.001
	> 91.11	3	68	95.77%		
Maximum bladder capacity (ml)	≤ 324.84	25	46	64.79%	3.858	0.050
	> 324.84	15	58	79.45%		
Qmax (ml/s)	≤ 7.05	22	50	69.44%	0.554	0.457
	> 7.05	18	54	75.00%		
Unstable contractions of bladder	NO	13	83	86.46%	29.094	<0.001
	YES	27	21	43.75%		

### Construction and validation of the multivariate logistic regression model

**Fig. 2:**
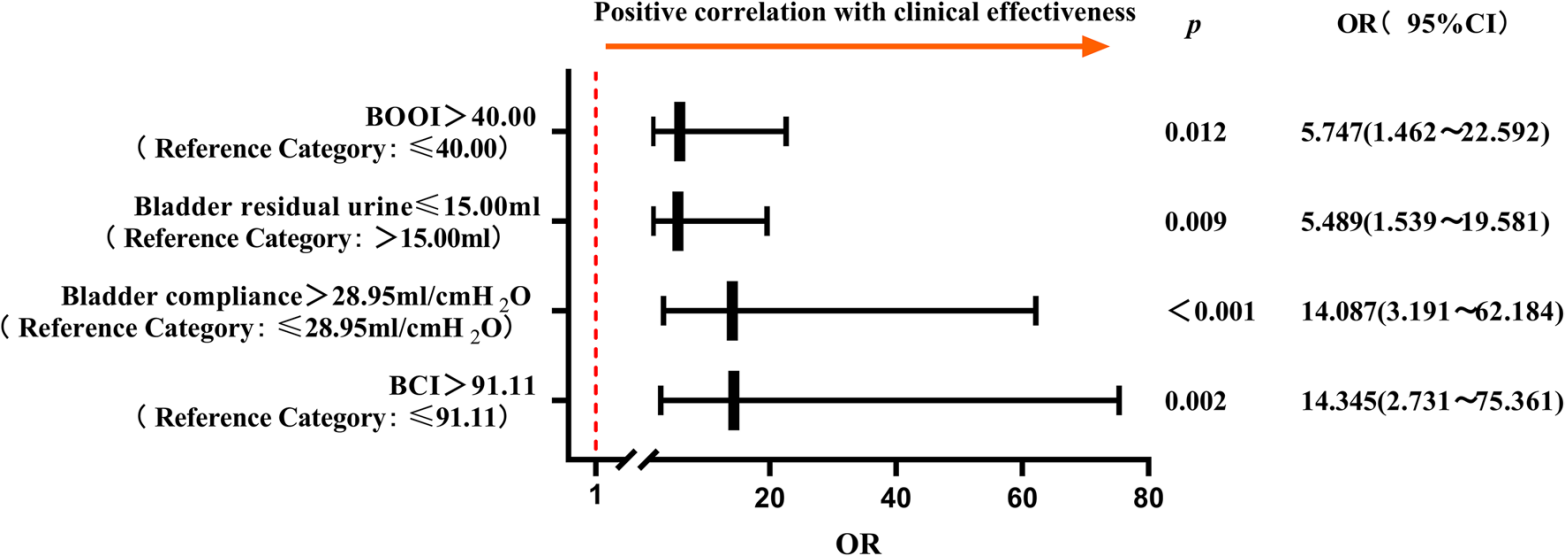
Forest plot analysis of urodynamic factors affecting the efficacy of TURP surgery

**Fig. 3:**
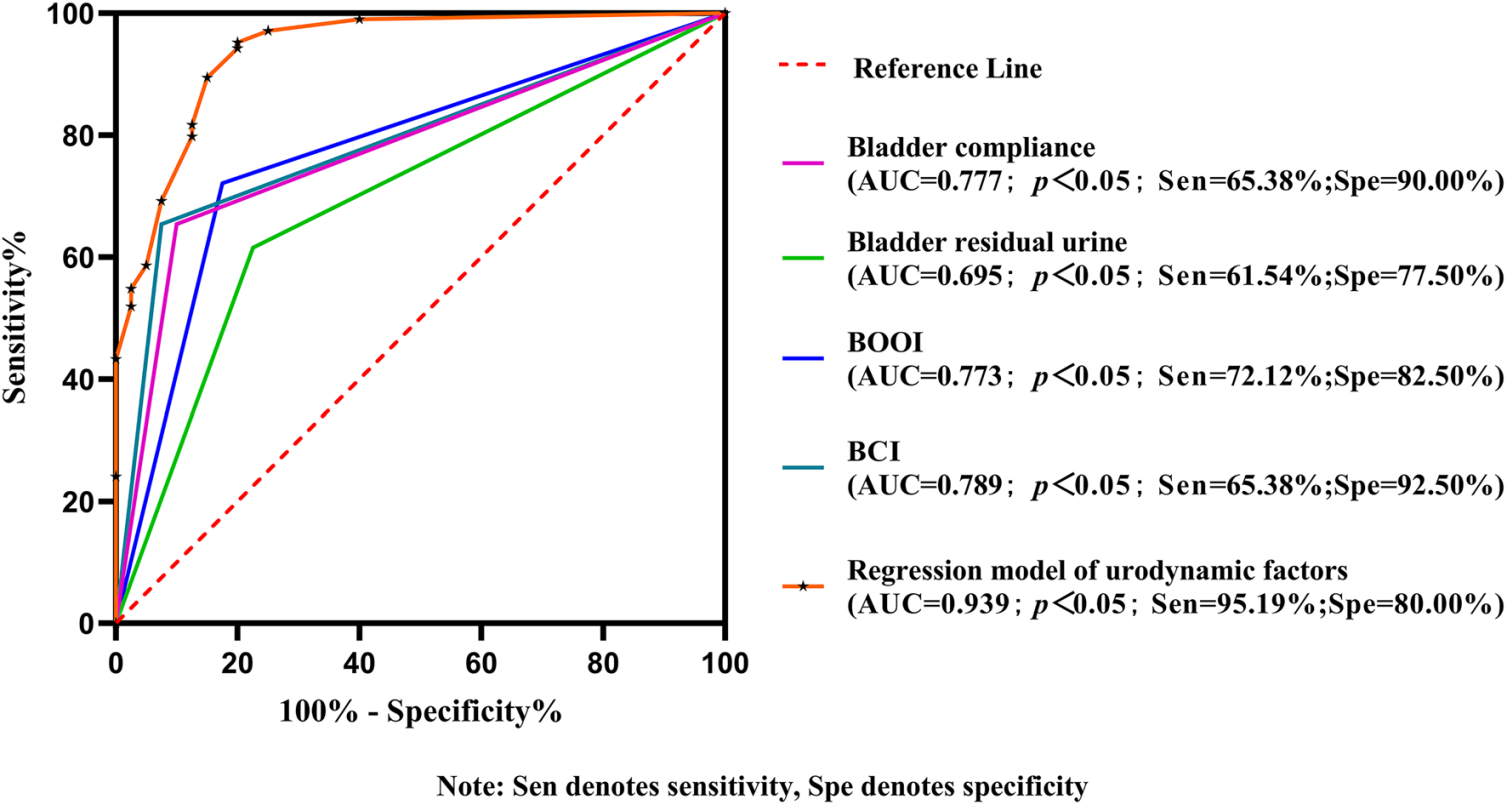
ROC curve analysis of the regression model

Factors considered to be statistically significant in the univariate analysis (*p* < 0.05) were included in the multifactor logistic regression analysis, for which the results are shown in Table 2. BCI (OR = 14.345, 95% CI 2.731–75.361, *p* = 0.002); BOOI (OR = 5.747, 95% CI 1.462–22.592, *p* = 0.012); bladder residual urine (OR = 5.489, 95% CI: 1.539–19.581, *p* = 0.009); and BC (OR = 14.087, 95% CI 3.191–62.184, *p* < 0.001) were independent influence factors in the efficacy of TURP surgery (Table 1; Fig. 2). The omnibus test showed *Χ*^2^ = 99.827, *p* < 0.001. The Hosmer–Lemeshow test showed *Χ*_hl_^2^ = 3.251, *p* = 0.918. ROC analysis of the regression model revealed that the regression model AUC = 0.939 (95% CI 0.886–0.972), with a sensitivity and specificity of 95.19% and 80%, respectively (Fig. 3).

**Table 2:** Urodynamic factors affecting the efficacy of TURP surgery (multivariate analysis)

		B	S.E	Wald	Sig	OR	95% C.I	
							Lower	Upper
BCI	> 91.11	2.663	0.846	9.903	0.002	14.345	2.731	75.361
	≤ 91.11	0				1		
BOOI	> 40	1.749	0.698	6.268	0.012	5.747	1.462	22.592
	≤ 40	0				1		
Bladder residual urine (ml)	≤ 15	1.703	0.649	6.886	0.009	5.489	1.539	19.581
	> 15	0				1		
Bladder compliance (ml/cmH_2_O)	> 28.95	2.645	0.758	12.193	<0.001	14.087	3.191	62.184
	≤ 28.95	0				1		
Unstable contractions of the bladder	NO	1.302	0.669	3.787	0.052	3.678	0.991	13.657
	YES	0				1		
Constants		-3.029	0.684	19.593	0	0.048

## Discussion

Factors influencing the outcome of TURP surgery in patients with BPH has been a hot topic for urologists for a long time [9–12]. Urodynamic analysis has been applied by some researchers in the study of factors affecting the outcome of TURP surgery due to its ability to make an accurate determination of bladder outlet obstruction and impaired bladder function caused by BPH. In recent years, different investigators have studied the relationship between different urodynamic parameters and the outcome of TURP surgery, and found that several urodynamic parameters, such as BOOI, DU, and detrusor instability, were associated with the outcome of TURP surgery. Most of these studies have explored the relationship between individual urodynamic parameters and surgical outcomes; however, the combined effect of multiple urodynamic parameters on surgical outcomes had not been fully investigated [13–16]. In addition, most of these studies were not randomized studies, and the statistical analysis results may be influenced by confounding factors, which would lead to conflicting results [17–21].

Our study included multiple urodynamic parameters and found that the following factors—BCI, BOOI, bladder residual urine, and BC—had independent effects on the outcome of TURP surgery according to logistic regression analysis, which reduced the effect of confounding factors. Our study also established a multifactor regression model that included multiple urodynamic indices for determining the surgical efficacy of TURP, which no similar study has reported before, to our knowledge.

Based on the ROC analysis, we found that when applied individually, each independent urodynamic factor is effective in determining surgical outcome; however, when used individually, each factor was less efficient than when combined, according to the regression model that combined all factors, thus suggesting that the regression model has a higher diagnostic efficiency in determining the efficacy of TURP surgery and deserves further investigation.

Several studies have constructed regression models that can predict the efficacy of BPH surgery. For example, Li et al. [22] found that patient age, BMI, duration of history of LUTS, and prostate volume were independent influences for postoperative urge incontinence through logistic regression models. Ye Tian et al. [23] reported that the P.R.OS.T.A.T.E. regression model could predict the outcome of BPH surgery in 2022. The main predictors of this model included age, IPSS score, length of prostatic intravesical protrusion, bladder wall thickness, thicknesses of the peripheral prostatic zone and migrating zone. This regression model had a diagnostic sensitivity of 70.6% and specificity of 75.6%. Few regression models include multiple urodynamic indices to diagnose the efficacy of TURP surgery. In our study, we developed a regression model with urodynamic parameters as the main independent influencing factors. The sensitivity and specificity of this regression model for diagnosing the efficacy of TURP surgery were 95.19% and 80%, respectively. Since urodynamic results directly reflect the functional changes of the lower urinary tract caused by BPH, it can be concluded that regression models based on urodynamic factors have a high value in predicting functional outcomes such as surgical outcomes.

However, the present study has some limitations, such as the small number of samples, which may affect statistical efficiency. Moreover, this study is a single-center study, and the regression model obtained lacks validation by external data of other centers. A multi-center study with a larger sample size is needed to explore this topic in depth.

Our findings suggest that BCI, BOOI, bladder residual urine, and BC have independent effects on the efficacy of TURP surgery. The regression model includes multiple urodynamic indices and thus has a higher diagnostic sensitivity and specificity in determining the efficacy of TURP surgery than using individual factors.

## Data Availability

The data used in this article have been published in sigshare, the DOI is 10.6084/m9.figshare.23821374
